# THE ROLE OF DIGESTIVE MANIFESTATIONS IN COVID-19 MORTALITY: EVIDENCES FROM BRAZIL’S FIRST EPIDEMIC WAVE

**DOI:** 10.1590/S0004-2803.24612025-061

**Published:** 2025-12-01

**Authors:** Elvis Paim FERREIRA, Mariana da Silva ARBUÉS, André Castro LYRA, Lourianne Nascimento CAVALCANTE

**Affiliations:** 1 Faculdade de Medicina da Bahia - UFBA; Salvador, BA, Brasil.; 2 Escola Bahiana de Medicina e Saúde Pública; Salvador, BA, Brasil.; 3 Hospital São Rafael - Rede D’Or São Luiz, Salvador, BA, Brasil.

**Keywords:** COVID-19, gastrointestinal manifestations, hepatology, healthcare disparities, Brazil, COVID-19, manifestações gastrointestinais, hepatologia, disparidades em saúde, Brasil

## Abstract

**Context:**

COVID-19, caused by SARS-CoV-2, is primarily characteri­zed by respiratory symptoms but also significantly affects the gastrointestinal (GI) tract and liver. Emerging evidence suggests that GI and hepatic manifestations may influence disease severity and outcomes. In Brazil, where disparities between public and private healthcare systems are pronounced, understanding these associations is crucial for optimizing patient management. This study aimed to evaluate the frequency and prognostic implications of digestive and hepatic injuries in hospitalized COVID-19 patients.

**Objective:**

To analyze the frequency of hepatic and gastrointestinal injuries and their association with outcomes (discharge or death).

**Methods:**

A retrospective cross-sectional study analyzed 3,555 patients from 50 private hospitals in Brazil (March-December 2020). Inclusion criteria were age ≥18 years, RT-PCR-confirmed COVID-19, and written consent. Demographic data, comorbidities, GI symptoms (diarrhea, nausea/vomiting, abdominal pain), liver enzymes (ALT, AST), and outcomes (discharge, mortality, ICU admission) were collected via REDCap. Statistical analyses included logistic regression to identify mortality predictors.

**Results:**

Among 3,555 patients (59.9% male, mean age 55.8 years). A total of 42.2% of patients presented with at least one gastrointestinal (GI) symptom at admission. Diarrhea was reported in 15%, nausea or vomiting in 13.6%, and abdominal pain in 6.5%, with symptom overlap among patients. The deceased group exhibited significantly higher alanine aminotransferase (ALT) (*p*=0.019) and aspartate aminotransferase (AST) (*p*=0.026) levels, representing 4.2- and 8.4-fold increases, respectively, with a higher AST/ALT ratio in fatal cases (1.69 vs 0.84). COVID-19 patients in Brazilian private hospitals showed hepatic abnormalities (AST 354.1 IU/L vs 42.1 IU/L in deceased vs discharged patients, *P*=0.026), with diarrhea associated with lower mortality (OR 0.61; 95%CI 0.42-0.88), while chronic liver disease (OR 2.95; 95%CI 1.35-6.41) and hypoalbuminemia (OR 0.22; 95%CI 0.11-0.38) emerged as independent predictors of death.

**Conclusion:**

Hepatic abnormalities and gastrointestinal symptoms are important markers of COVID-19 severity. Diarrhea, liver enzyme alterations, and serum albumin levels were significant predictors of mortality. Future strategies should prioritize hepatic monitoring, nutritional support, and healthcare equity through targeted interventions for high-risk groups, particularly in resource-limited settings.

## HIGHLIGHTS

• To analyze the frequency of hepatic and gastrointestinal injuries and their association with outcomes (discharge or death).

• Cross-sectional study involving 3555 patients across 50 private hospitals in Brazil.

• Diarrhea, abnormal liver enzymes, and hypoalbuminemia emerged as significant mortality predictors in COVID-19 patients treated at Brazilian private hospitals.

## Resumo

**Contexto:**

A COVID-19, causada pelo SARS-CoV-2, é caracterizada principalmente por sintomas respiratórios, mas também afeta significativamente o trato gastrointestinal (GI) e o fígado. Evidências recentes sugerem que manifestações GI e hepáticas podem influenciar a gravidade e os desfechos da doença. No Brasil, onde as disparidades entre os sistemas de saúde público e privado são acentuadas, compreender essas associações é crucial para otimizar o manejo dos pacientes. Este estudo teve como objetivo avaliar a frequência e as implicações prognósticas das lesões digestivas e hepáticas em pacientes hospitalizados com COVID-19.

**Objetivo:**

Analisar a frequência de lesões hepáticas e gastrointestinais e sua associação com desfechos (alta ou óbito).

**Métodos:**

Estudo transversal retrospectivo que analisou 3.555 pacientes de 50 hospitais privados do Brasil (março-dezembro/2020). Os critérios de inclusão foram idade ≥18 anos, confirmação de COVID-19 por RT-PCR e consentimento por escrito. Dados demográficos, comorbidades, sintomas GI (diarreia, náuseas/vômitos, dor abdominal), enzimas hepáticas (ALT, AST) e desfechos (alta, mortalidade, admissão em UTI) foram coletados via REDCap. Análises estatísticas incluíram regressão logística para identificar preditores de mortalidade.

**Resultados:**

Dos 3555 pacientes (59,9% homens, média de idade de 55,8 anos), 42,2% apresentaram sintomas GI na admissão, sendo os mais comuns: diarreia (35,6%) e náuseas/vômitos (32,4%). O grupo de óbito apresentou níveis mais elevados de alanina aminotransferase (ALT) (*P*=0,019) e aspartato aminotransferase (AST) (*P*=0,026), representando aumentos de 4,2 e 8,4 vezes, respectivamente; sendo a razão AST/ALT maior nos casos fatais (1,69 vs 0,84). Os pacientes hospitalizados por COVID-19 em hospitais privados brasileiros apresentaram alterações hepáticas (AST 354,1 vs 42,1 em pacientes falecidos vs que obtiveram alta, *P*=0,026), com diarreia associada a menor mortalidade (OR 0,61; IC95% 0,42-0,88) e doença hepática crônica (OR 2,95; IC95% 1,35-6,41) e hipoalbuminemia (OR 0,22; IC95% 0,11-0,38) como preditores independentes de óbito.

**Conclusão:**

Alterações hepáticas e sintomas gastrointestinais são marcadores importantes de gravidade na COVID-19. Diarreia, alteração de enzimas hepáticas e albumina sérica foram importantes preditores de mortalidade. Estratégias futuras devem priorizar monitoramento hepático, suporte nutricional, e equidade no sistema de saúde por meio de intervenções direcionadas a grupos de alto risco, especialmente em locais com recursos limitados.

## INTRODUCTION

The pandemic caused by SARS-CoV-2 has resulted in over 630 million confirmed cases and 6 million deaths globally^1^. In Brazil, there have been over 35 million confirmed cases and more than 680,000 deaths^1^. The primary symptoms of SARS-CoV-2 infection are fever, cough, and dyspnea^2^ ^,^ ^3^. However, COVID-19 can also affect the digestive system, with recognized gastrointestinal symptoms including diarrhea, anorexia, nausea, vomiting, and abdominal pain^2^ ^-^ ^6^. Diarrhea is the most common, with prevalence varying from 1% to 36% across studies^2^ ^-^ ^4^. Anorexia and nausea have also been frequently observed, with reported prevalences of up to 17.9%^2^ ^,^ ^3^.

Additionally, COVID-19 can lead to alterations in liver enzyme levels, suggesting hepatic involvement, and is associated with an incidence of 58-78% among fatal cases^7^. In severe cases, some patients have experienced liver failure or have undergone liver transplantation^3^ ^,^ ^4^. The detection of SARS-CoV-2 RNA in fecal samples suggests that the gastrointestinal tract is a site of infection and a potential transmission route^2^ ^,^ ^8^. Gastrointestinal symptoms may correlate with a more extended clinical course and, in some cases, influence prognosis, as observed in certain studies^6^ ^,^ ^8^. However, variability in data indicates further research is needed to comprehend their impact fully.

The spread of the virus and the pandemic’s effects significantly varied, influenced by numerous economic, political, and social factors. Socio-spatial inequalities played a significant role in shaping the outcomes of the COVID-19 pandemic, with differences in healthcare access being particularly impactful. Studies have shown variation in hospital performance based on legal status (private vs public), with evidence such as higher mortality rates among patients admitted to public hospitals in Brazil^9^ ^-^ ^12^.

It is important to determine the frequency of gastrointestinal and liver complications in COVID-19 patients to assess the need for individualized therapy, which may improve prognosis. Identifying key variables related to patient outcomes, including differences between public and private hospitals, can enhance early diagnosis and prognosis, ultimately leading to more effective treatment strategies, especially in anticipation of potential new waves of infection. Therefore, the primary objective of this study was to evaluate the frequency of gastrointestinal and liver injuries among patients hospitalized with COVID-19 in private hospitals in Brazil and its possible association with outcomes (discharge and death).

## METHODS

This study is a retrospective, cross-sectional analysis of patients hospitalized with SARS-CoV-2 infection at various private hospitals in Brazil between March and December 2020. A total of 50 private hospitals participated in this research, and we selected patients from these facilities. To ensure data quality and consistency, all clinical and laboratory information was collected using a standardized electronic case report form (REDCap). Participants included in the study were required to be over 18 years old and a confirmed COVID-19 diagnosis, verified by reverse transcription polymerase chain reaction (RT-PCR) testing for SARS-CoV-2. Individuals who did not provide written informed consent were excluded from the study. The SARS-CoV-2 diagnostic test, performed using nasopharyngeal swabs or saliva samples, showed a sensitivity of 86% (95% Confidence Interval [CI]: 84-88%) and a specificity of 96% (95%CI: 94-97%), resulting in a positive likelihood ratio of 18.8 (95%CI: 14.5-24.3). Sociodemographic and clinical information was collected from medical records across all 50 private hospitals using Research Electronic Data Capture (REDCap) forms. The research team documented clinical, laboratory, and procedural data regarding patient admission, discharge, or mortality.

Information on the criteria for intensive care unit (ICU) admission and subsequent outcomes (hospital discharge or death) were also recorded. Reported digestive symptoms were assessed during hospital admission, and relevant laboratory tests were performed at that moment and before ICU admission. The study outcomes were defined as follows: discharge with improved health conditions, death, transfer to another hospital (as requested by the patient or their relatives), extended hospitalization, and palliative care.

### Statistical analysis

For normally distributed continuous variables, data are reported as mean ± standard deviation (SD), whereas skewed variables are summarized with the median and interquartile range. Categorical variables are expressed as frequencies and percentages. The Shapiro-Wilk test was used to evaluate the normality of continuous data. Quantitative comparisons were performed using the Student’s T-test or, when appropriate, the nonparametric Mann-Whitney U test, while categorical variables were compared using the chi-squared (χ²) test or Fisher’s exact test. All tests were two-tailed, and a *p*-value of less than 0.05 was considered statistically significant. To identify independent predictors of COVID-19 mortality, we performed multivariable logistic regression incorporating variables with *p*<0.2 from bivariate analyses (chi-square tests for categorical variables; Mann-Whitney U/t-tests for continuous variables), excluding collinear predictors (VIF<5), and assessed model fit using Hosmer-Lemeshow goodness-of-fit test and area under the ROC curve. All statistical analyses were performed conducted with SPSS version 23.0 (IBM, United States).

### Ethical aspects

The Institutional Review Board (IRB) of each private hospital approved the study (CAAE: 29496920.8.0000.5262). It is part of the secondary arm of the research titled “Protocolo de Caracterização Clínica para Infecções Emergentes Severas do ISARIC/OMS: Coronavírus,” which is being conducted by the Instituto D’Or de Pesquisa e Ensino. Our research team independently performed the analysis without any financial support, and we had consent to access the IDOR database. All patients provided written informed consent explaining that participation was voluntary and that there would be no penalties for choosing not to participate at any time. The study adhered to good clinical practices in research and followed the guidelines outlined in the 1975 Declaration of Helsinki and its subsequent amendments. There were no ethical conflicts associated with this study.

## RESULTS

### Demographic and clinical characteristics

Our study analyzed 3,555 COVID-19 patients treated at private hospitals in Brazil. The cohort showed a predominance of male patients, with 59.9% (n=2,129) being male and a mean age of 55.8 (17.1) years. The age distribution of the participants was as follows: 60.9% were under 60 years, 29.3% were aged 60-79 years, and 9.7% were 80 years or older. At the time of admission, 39.6% (n=1,409) of the patients presented with hypoxemia (SpO_2_ < 94%), and 6.3% (n=223) required immediate invasive ventilation. Additionally, 78.8% (n=2,803) required intensive care unit (ICU) care during hospitalization, indicating the severity of the study population (Table 1). Among the patients included in the study, 87.1% (n = 3,095) were discharged from the hospital, while 10.4% (n=369) died, 0.3% (n=10) were discharged to receive palliative care at home, and 1.8% (n=64) remained hospitalized for a prolonged period. Among these patients, 49.6% (n=1,762) had arterial hypertension, 26.3% (n=935) had type 2 diabetes mellitus (DM2), 22.4% (n=796) were obese, and 1.1% (n=38) had a history of chronic liver disease (Table 1). Regarding comorbidities, 32.6% had only one condition, 7.9% had two conditions, and 0.5% had three or more conditions (Table 1). Regarding respiratory symptoms related to COVID-19, 41.4% (n=1,471) of the patients experienced dyspnea upon arrival at the hospital. Before admission, 10.8% of patients (n=385) had taken hydroxychloroquine, but without any association with clinical outcomes. Other COVID-19 characteristics of patients were reported in Table 1.

**TABLE 1:** Demographic and clinical characteristics of COVID-19 patients upon hospital admission (N=3555)

	Characteristics	N (%)
Sex	Male	2129 (59.9)
	Female	1424 (40.1)
	No information	2 (0.1)
Age	Mean (SD), Years	55.8 (17.1)
ICU Admission	Yes	2803 (78.8)
	No	710 (20.0)
	No information	42 (1.2)
Peripheral oxygen saturation	<=94%	1409 (39.6)
	>94%	1712 (48.2)
	No information	434 (12.2)
Invasive ventilation	Yes	223 (6.3)
	No	3254 (91.5)
	No information	18 (1.7)
Number of Comorbidities*	None	2096 (59.0)
	1	1158 (32.6)
	2	282 (7.9)
	3 or more	19 (0.5)
Obesity	Yes	796 (22.4)
	No	2643 (74.3)
	No information	116 (3.3)
Previous chronic liver disease	Yes	38 (1.1)
	No	3472 (97.7)
	No information	45 (1.3)
Type 2 Diabetes	Yes	935 (26.3)
	No	2562 (72.1)
	No information	58 (1.6)
Arterial hypertension	Yes	1762 (49.6)
	No	1727 (48.6)
	No information	66 (1.9)
Gastrointestinal symptoms	Diarrhea	534 (15.0)
	No Diarrhea	2940 (82.7)
	No information	81 (2.3)
	Abdominal pain	232 (6.5)
	Nausea or Vomiting	485 (13.6)
	Ageusia	248 (7.0)
Respiratory symptoms	Chest pain	240 (6.8)
	Dyspnea	1471 (41.4)
	Cough	1755 (49.4)
	Sore throat	254 (7.1)
Other symptoms	Headache	614 (17.3)
	Myalgia	943 (26.5)
	Confusion	205 (5.8)
	Seizures	30 (0.8)
	Rash	24 (0.7)
	Enlarged lymph nodes	1 (0.0)
Therapy (before admission)	Hydroxychloroquine treatment	385 (10.8)

Notes: *obesity. diabetes. malnutrition. chronic liver disease. Categorical variables are expressed as N (%). DM2. Diabetes mellitus type 2; ICU. intensive care unit; SaO2. arterial oxygen saturation. Patients may have presented with more than one gastrointestinal symptom; reported percentages are not mutually exclusive.

### Gastrointestinal and hepatic manifestations

At admission, a significant proportion of patients (n=1,499) presented with gastrointestinal symptoms. Diarrhea was reported in 534 individuals (15.0%), while 2.3% of patients had no recorded information regarding this symptom. In addition, abdominal pain was observed in 232 patients (6.5%), and nausea or vomiting was reported by 485 patients (13.6%). Ageusia was noted in 248 subjects (7.0%). Although these gastrointestinal manifestations were less prevalent than some respiratory symptoms (with dyspnea noted in 41.4% of patients and cough in 49.4%), the occurrence of diarrhea and nausea or vomiting underscores the importance of assessing digestive tract involvement in the clinical evaluation (Table 1). These symptoms were not mutually exclusive, and many patients reported more than one gastrointestinal manifestation simultaneously.

Liver enzyme abnormalities were widespread among the patients. The average levels were as follows: AST: 68.8 (+397.6) IU/L (range: 2-17,007), ALT: 68.4 (+138.6) IU/L (range: 3-5,175), and alkaline phosphatase: 87.0 (+62.3) IU/L. Serum albumin levels of 3.5 (+6.5)g/dL, and elevated D-dimer levels were observed, averaging 1,665 (+6,533.7) ng/mL, with 36.3% of patients exceeding 1,000 ng/mL. The average platelet count was 257.6 (± 187.5) x 10^3^/µL (range: 2-14400 x 10^3^/µL).

### Endoscopic findings

A total of 41 patients underwent 53 upper gastrointestinal endoscopies (EGDs) and 7 colonoscopies during their hospitalization. There was a slight male predominance (22 patients, 53.6%) and the average age was 68.6 years, 7-98 years). Of the 41 patients evaluated, 36 (87.8%) were admitted to intensive care units and 31 (75.6%) required invasive ventilatory support during hospitalization. Anticoagulant therapy was administered to 35 (85.3%) patients, with 20 (57.1%) receiving prophylactic doses, seven (20%) receiving therapeutic doses, and eight (22.8%) receiving extended doses (enoxaparin 40 mg every 12 hours). The main indication for upper gastrointestinal endoscopy was a drop in hemoglobin levels (23 patients, 43.3%), followed by the placement of a nasoenteric tube (14 patients, 26.4%). Conversely, of the seven colonoscopies performed, six (85.7%) were indicated for lower gastrointestinal bleeding and one (14.3%) for decompressive colonoscopy in a patient with colonic pseudo-obstruction.

Regarding the primary endoscopic findings from upper gastrointestinal endoscopies, erosive and erythematous gastritis were observed in 22 (41.5%) procedures, followed by erosive duodenitis in nine (16.9%), two of which exhibited active bleeding, necessitating endoscopic therapeutic intervention with an epinephrine injection in one instance. Gastric ulcers were noted in seven (13.2%) examinations, with five being active; epinephrine injection was chosen for one case due to the presence of an adherent clot. Duodenal ulcers were noted in six (11.3%) endoscopies, with active bleeding evidenced in four and endoscopic therapy performed in three, including epinephrine injection in two cases and a combination of hemoclip and epinephrine injection in one. The primary findings during colonoscopy included diverticular disease and hemorrhoidal disease, found in two (28.6%) examinations, followed by terminal ileal ulcers in one (14.3%) examination, and the presence of polyps, vascular ectasias, and diffuse distension of the colon and rectum, indicative of Ogilvie syndrome. No rebleeding occurred after any of the instituted endoscopic therapies, and 23 patients succumbed to complications related to COVID-19. None of the endoscopists acquired SARS CoV 2 infection during the study period.

### Outcome predictors

Regarding clinical symptoms, abdominal pain was reported in 89.2% of discharged patients versus 10.8% of patients who died, with the *P*-value of 0.297 and an OR of 0.88 (95%CI: 0.58-1.37) indicated no statistically significant association between abdominal pain and the outcomes. Diarrhea was reported in 93.0% of discharged patients, compared to only 7.0% of those who died (P = 0.008; OR = 0.63; 95% CI: 0.44-0.89). This symptom was significantly associated with favorable outcomes, suggesting that patients presenting with diarrhea were more likely to be discharged than those without it (Table 2).

**TABLE 2:** Bivariate analysis of clinical variables and patient outcomes (N=3464).

Predictor	Discharge	Death (n/%)	***P*-value**	OR (Death)	95%CI
Abdominal pain n (%)	207 (89.2%)	25 (10.8%)	0.297	0.88	0.58-1.37
Diarrhea n (%)	1401 (93.0%)	37 (7.0%)	0.008	0.63	0.44-0.89
ICU admission n (%)	2364 (86.9%)	366 (13.1%)	<0.001	33.33	11.11-100.0
Obesity n (%)	700 (90.2%)	76 (9.8%)	0.428	1.12	0.86-1.47
Malnutrition n (%)	20 (66.7%)	10 (33.3%)	0.055	4.46	1.11-17.89
Type 2 Diabetes n (%)	692 (75.2%)	228 (24.8%)	<0.001	2.92	1.90-4.50
Chronic liver disease n (%)	27 (75.0%)	9 (25.0%)	0.011	2.84	1.33-6.09
Number of Comorbidities* (n) %					
1	983 (87.3)	143 (12.7)	<0.001		
2	228 (85.1)	40 (14.9)			
3 or more	12 (66.7)	6 (33.3)			
Male gender n (%)	1868 (87.5%)	268 (12.5%)	0.172	1.19	0.93-1.51
O_2_ saturation <94% n (%)	1194 (85.0%)	211 (15.0%)	0.001	1.67	1.24-2.24
Age (years) mean (SD)	55.8	55.9	0.070		
ALT (IU/L) mean (SD)	49.9 (44.3)	210.0 (769.9)	0.019		
AST (IU/L) mean (SD)	42.1 (33.5)	354.1 (1583.2)	0.026		
Albumin (g/dL) mean (SD)	3.7 (0.8)	2.2 (0.5)	<0.001		
Platelets, (x10^3^) mean (SD)	217.9(88.7)	936.6 (13444.2)	0.324		
Bilirrubin, mg/dL mean (SD)	8.0 (4.3)	5.3 (0)	0.600		
Alkaline phosphatase (IU/L) mean (SD)	146.7 (12.7)	267.3 (44.3)	0.010		
Creatinine mg/dL mean (SD)	1.2 (0.2)	2.4 (0.2)	<0.001		
Hemoglobin g/dL mean (SD)	13.1 (0.1)	9.8 (0.3)	<0.001		
D-dimer (ng/mL) mean (SD)	1199.6 (3682.7)	3494.5 (12788.8)	0.017		

Notes: This table includes absolute and percentage values. It was considered as significant *P*-value <0.05. Total N after excluding cases with missing outcome data: 3464. *Obesity, Diabetes, Malnutrition, Chronic Liver Disease. OR=Odds Ratio; CI=Confidence Interval; AST aspartate aminotransferase; ALT alanine aminotransferase; ICU Intensive Unit Care.

A greater number of comorbidities was associated with increased mortality risk in a dose-dependent manner, despite small sample size in the ≥3 comorbidities group.

In terms of pre-existing clinical conditions and nutritional status, obesity was present in 90.2% of discharged patients and 9.8% of those who died (*P*=0.428; OR 1.12, 95%CI: 0.86-1.47), indicating no statistically significant relationship with outcomes. Malnutrition, evaluated in a smaller subset (n=30), was identified in 66.7% of discharged patients and 33.3% of those who died; and, although the *P*-value was marginal (*P*=0.055), the OR of 4.46 (95%CI: 1.11-17.89) suggests that malnourished patients had over four times chance of an adverse outcome. The type 2 diabetes was recorded in 75.2% of discharged patients versus 24.8% of patients who died, with a statistically significant *P*-value of <0.001 and an OR of 2.92 (95%CI: 1.89-4.50). Pre-existing chronic liver disease was observed in 75.0% of discharged patients compared to 25.0% of those who died (*P*=0.011; OR 2.84, 95%CI: 1.33-6.09). The analysis also revealed that an increasing number of comorbidities was linked to a higher proportion of deaths (12.7% for one comorbidity, 14.9% for two, and 33.3% for three or more; *P*<0.001), highlighting the impact of comorbidity burden on prognosis (Table 2).

**FIGURE 1:**
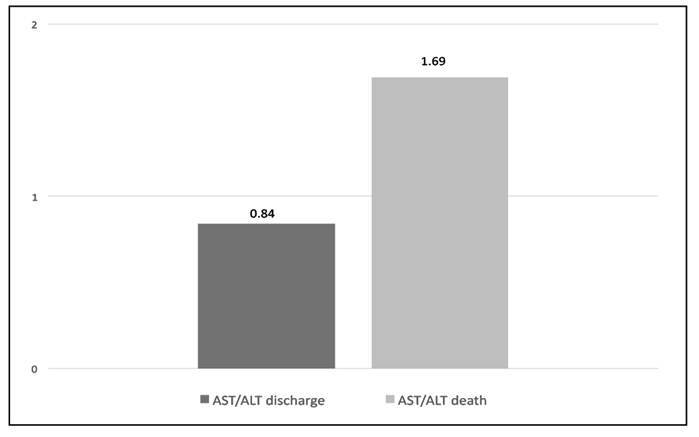
Comparison of AST/ALT Ratios in COVID-19 survivors versus non survivors.

Considering laboratory tests, hepatic enzyme levels exhibited significant differences between the discharge and death groups (Table 2). In patients who died, the mean alanine aminotransferase (ALT) level was 210.0 IU/L compared to 49.9 IU/L in discharged patients (*P*=0.019). Similarly, aspartate aminotransferase (AST) levels were significantly higher in the death group (354.1 IU/L) compared with those in the discharged group (42.1 IU/L, *P*=0.026). It is important to emphasize the relative magnitude of these elevations: ALT was approximately 4.2 times higher in the death group, whereas AST demonstrated an even more pronounced increase, being about 8.4 times greater than in the discharge group. The ratio of aspartate aminotransferase (AST) to alanine aminotransferase (ALT) between outcome groups was performed: among discharged patients, the AST/ALT ratio was 0.84 and, among patients who died, the AST/ALT ratio was 1.69 (Figure 1).

The elevated AST/ALT ratio observed in non-survivors may indicate mitochondrial injury or extrahepatic involvement, such as muscle damage, consistent with severe systemic inflammatory conditions. As a widely accessible biomarker, this ratio could aid in the initial risk stratification of hospitalized patients.

Serum albumin levels were significantly lower in the death group (2.2 g/dL vs 3.7 g/dL, *P*<0.001). Alkaline phosphatase levels were also significantly elevated in the death group (267.3 vs 146.7 UL, *P*=0.010) (Table 2). Bilirubin and the international normalized ratio (INR) showed no significant differences between groups (*P*=0.600 and *P*=0.901, respectively).

The mean D-dimer level was significantly higher among those who died (3494.5 ng/mL vs 1199.6 ng/mL; *P*=0.017). Renal function, as measured by creatinine, was significantly worse in patients who died (2.4 mg/dL vs 1.2 mg/dL; *P*<0.001), and hemoglobin levels were also significantly lower in the death group (9.8 g/dL vs 13.1 g/dL; *P*<0.001), possibly reflecting a deteriorated clinical status (Table 2).

Patients admitted to the ICU had a discharge rate of 87%, and 13.1% died and this difference was highly significant (*P*<0.001), with an OR of 33.3. The proportion of people with O_2_ saturation ≤94% was 85% in the discharged group compared to 15% in the death group (OR 1.67, *P*=0.001) (Table 2). With respect to demographic and clinical variables, male gender did not show a statistically significant association with the outcomes (87.5% in the discharge group vs 12.5% in the death group (OR 1.19, *P*=0.172). Similarly, age (analyzed as a continuous variable per year increased) was not significantly associated with outcomes (*P*=0.070) (Table 2).

The multivariable logistic regression analysis identified several independent predictors of mortality among non-collinear variables. The presence of diarrhea was inversely associated with mortality (β= -0.50; adjusted OR, 0.61; 95%CI, 0.42-0.88; *P*=0.007), suggesting that patients presenting with diarrhea experienced a 39% reduction in the odds of death. Regarding liver enzymes, each one-unit increase in ALT was associated with a 1.2% increase in the odds of death (adjusted OR=1.012; *P*=0.009). Preexisting chronic liver disease was a significant predictor, conferring nearly threefold higher chances of death (adjusted OR, 2.58; 95%CI, 1.35-6.41; *P*=0.010). Notably, serum albumin was identified as an exceptionally robust protective factor; every 1 g/dL increase in albumin was associated with a 78% reduction in the odds of death (adjusted OR=0.22; 95%CI, 0.11-0.38; *P*<0.001). Among demographic variables, age (per year increase) was not significantly associated with the outcome (adjusted OR ≈1.00; 95%CI, 0.98-1.02; *P*=0.072), and male sex did not reach statistical significance (adjusted OR, 1.22; 95%CI, 0.94-1.59; *P*=0.150). Additionally, for every 1000 ng/mL increase in D-dimer, the chance of death increased by 36% (adjusted OR=1.36; 95%CI, 1.07-1.74; *P*=0.015). ICU admission demonstrated a pronounced effect (adjusted OR 35.45; 95% CI 11.85-106.20), indicating that patients requiring intensive care had markedly higher odds of death compared with those not admitted (Table 3).

**TABLE 3:** Multivariable logistic regression model to identify independent predictors of death among COVID-19 patients.

Predictor	OR	95% CI	z	*P*	OR	95%CI	z	*P*	VIF
	Crude	Crude	Crude	Crude	Adjusted	Adjusted	Adjusted	Adjusted	
ICU admission	33.33	11.11 -100.00	5.92	<0.001	35.45	11.85 -106.20	6.01	<0.001	1.12
Chronic liver disease	2.84	1.33 - 6.09	2.55	0.011	2.95	1.35 - 6.41	2.58	0.010	1.08
Albumin (per g/dL)	0.22	0.11 - 0.38	-4.73	<0.001	0.22	0.11 - 0.38	-4.75	<0.001	1.30
D-dimer (per 1000 ng/mL)	1.36	1.07 - 1.74	2.43	0.015	1.36	1.07 - 1.74	2.45	0.015	1.22
ALT (per IU/L)	1.012	1.004 - 1.021	2.63	0.009	1.012	1.004 - 1.021	2.65	0.009	1.18
Diarrhea	0.63	0.44 - 0.89	-2.72	0.007	0.61	0.42 - 0.88	-2.70	0.007	1.05
Age (per year)	1.001	0.98 - 1.02	1.81	0.072	1.001	0.98 - 1.02	1.80	0.072	1.15
Male sex	1.19	0.93 - 1.51	1.44	0.150	1.22	0.94 - 1.59	1.48	0.150	1.10

Results of the logistic regression analysis for the outcome “death”. Variables with *P*<0.2 in the bivariate analysis (TABLE 2) were considered. In this analysis, only non-collinear variables (VIF<5) were considered. Hosmer-Lemeshow test: *P*=0.62, AUC (ROC curve): 0.89, Nagelkerke R²: 0.52. Adj: adjusted results.

## DISCUSSION

This study examines hepatic and gastrointestinal manifestations of COVID-19 in a large Brazilian cohort (n=3,555) from private hospitals during the pandemic’s first wave (pre-vaccine era), revealing critical mortality predictors and stark healthcare disparities. By focusing on private institutions with greater resource availability, we established a comparator for evaluating structural inequalities in Brazil’s dual healthcare system while characterizing organ-specific pathology during this early, treatment-naïve period. Our findings both confirm global patterns of COVID-19-related hepatic injury and gastrointestinal involvement, and provide novel insights into socioeconomic determinants of outcomes through comparison with national SARS surveillance data.

The marked elevation of liver enzymes in non-survivors-particularly the 4.2-fold higher ALT (210.0 vs 49.9 IU/L) and 8.4-fold higher AST (354.1 vs 42.1 IU/L) -corroborates global reports of COVID-19-associated hepatocyte injury^7^ ^,^ ^13^. The AST/ALT ratio of 1.69 in fatal cases (vs 0.84 in survivors) suggests disproportionate mitochondrial injury or extrahepatic damage (e.g., cardiac, skeletal muscle), consistent with studies linking this ratio to multiorgan failure^14^ ^-^ ^16^. Notably, our multivariable model identified ALT elevation as an independent mortality predictor (aOR 1.012, *P*-value=0.009)^15^. These findings reinforce recommendations by Marjot et al.^13^ to monitor liver enzymes serially in hospitalized patients, as they reflect both direct viral cytopathy and systemic inflammation.

Higher serum albumin was strongly protective, whereas hypoalbuminemia was associated with increased mortality. Hypoalbuminemia (<2.2 G/dl in deceased) highlighting its role as a biomarker of he­patic dysfunction, malnutrition, or systemic inflam­mation. This aligns with Aktan et al.^17^ demonstration that nutritional scores predict covid-19 outcomes. The scientific literature reinforces the association between malnutrition and poor prognosis in patients with covid-19, showing that elevated malnutrition scores (conut and pni) are correlated with higher mortality. Additionally, adequate nutritional support has been shown to strengthen immunity, help prevent progression to severe cases, promote better recovery, and reduce complications^17^. These findings emphasize the importance of comorbidity-specific risk stratification and highlight the need for early, targeted nutritional support in hospitalized patients with covid-19.

The elevated mortality risk among patients with chronic liver disease (aOR 2.95, P=0.010) aligns with global findings, underscoring the critical importance of systematic screening for hepatic comorbidities in COVID-19 care^7^ ^,^ ^13^. The pre-existing liver disease has been associated with higher susceptibility to COVID-19-related hepatic decompensation and acute liver failure^7^ ^,^ ^13^. Studies have suggested two pathways of injury: (1) direct hepatocytic damage via SARS-CoV-2 replication (mediated by ACE2 receptor expression) and (2) secondary insults, including hypoxic damage, cytokine-mediated inflammation, and drug-induced liver injury, particularly in this high-risk population^13^. Other variables should be considered, including the systemic inflammatory response or hypoxia, use of hepatotoxic drugs, and pre-existing comorbidities^13^.

Diarrhea was the most prevalent gastrointestinal symptom, followed by nausea or vomiting, ageusia and abdominal pain. A meta-analysis by Alzahrani et al. (2022) identified nausea/vomiting as the most pervasive manifestation^18^ ^,^ ^19^. However, the frequency of symptoms found in this study was like that observed in other studies^18^ ^,^ ^19^. The observed protective association of diarrhea (aOR 0.61, 95%CI 0.42-0.88; *P*=0.007) contrasts with early pandemic reports of gastrointestinal involvement as a severity marker^3^ ^,^ ^6^, but aligns with more recent evidence from large meta-analyses^18^ ^,^ ^19^. Three non-exclusive mechanisms may explain this finding: (1) enhanced viral clearance through ACE2/TMPRSS2-mediated gut immune responses, as demonstrated by Yao et al.^5^ ^,^ ^18^; (2) earlier healthcare-seeking behavior among patients with gastrointestinal symptoms, leading to prompt medical attention^8^; and (3) SARS-CoV-2-induced alterations in gut microbiota composition that may mitigate systemic inflammation through the gut-lung axis^5^. Abdominal pain showed no mortality association (OR 0.88, *P*=0.297), suggesting it may arise from non-specific visceral hypersensitivity rather than severe pathology^4^. The receptors for SARS-CoV-2, angiotensin-converting enzyme 2 (ACE2), and the cellular serine protease required for viral entry, transmembrane serine protease 2 (TMPRSS2), are co-expressed in the cells of various gastrointestinal (GI) organs. These include cholangiocytes, colonocytes, esophageal keratinocytes, ileal absorptive enterocytes, and pancreatic β-cells. This suggests that direct viral injury is the primary cause of the GI symptoms associated with COVID-19^19^.

Data from the severe acute respiratory syndrome (SARS) surveillance system available on OpenDataSUS, have showed that, in Brazilian public and private hospitals, the most frequent gastrointestinal symptoms were 15.2% had diarrhea (n=14912; *P*=0.48) and 9.5% had vomiting (n=9209; *P*<0.001)^20^.

In our cohort, 10.8% of patients reported using hydroxychloroquine prior to hospital admission. Although hydroxychloroquine was widely utilized during the early stages of Brazil’s COVID-19 _ with one trial noting that only 7.5% of participants were HCQ-naïve^21^ -a subsequent large multicenter study reported its use in 9.0% of hospitalized cases (10.9% of non-survivors vs 8.5% of survivors)^22^. The relatively low rate of hydroxychloroquine use in our privately treated population likely reflects greater adherence to evolving evidence-based guidelines discouraging unproven therapies. Additionally, gastrointestinal symptoms observed in our study should be interpreted in the context of Brazil’s early treatment practices. A cross-sectional study found that individuals prescribed these off-label regimens reported significantly more abdominal pain, diarrhea, and fecal urgency than those who received no treatment, with lower-income groups disproportionately exposed to these medications^23^. However, their systematic review and network meta-analysis found no statistically significant association between the combination of hydroxychloroquine and azithromycin and symptoms such as nausea, vomiting, or abdominal pain when compared with controls or monotherapy. The authors emphasized the very low certainty of the available evidence and highlighted the need for robust randomized trials to clarify these safety outcomes^23^. In the present study, we did not evaluate the association between hydroxychloroquine use and reported gastrointestinal symptoms.

Endoscopic evaluation demonstrated erosive gastritis (41.5%) and gastric ulcers (13.2%) in the upper GI procedures, with diverticular disease (28.6%) detected on colonoscopy. These observations corroborate the global reports of COVID-19-associated mucosal injury in critical patients, where hypoxia, therapeutic anticoagulation, and physiological stress are implicated as pathogenic factors^24^ ^,^ ^25^. Notably, the lack of post-intervention rebleeding events supports the safety of endoscopic procedures in this cohort when adhering to standardized protocols^23^. These findings corroborate reports of COVID-19-associated mucosal injury likely due to hypoperfusion and anticoagulant use^26^.

ICU admission emerged a robust predictor of death, with an adjusted OR of 35.45, reinforcing that the need for intensive care is a critical marker of severity. This finding underscores the importance of monitoring patients who progress to severe conditions, requiring early interventions. Type 2 diabetes (crude OR: 2.92) remained significant predictor even after adjustment, emphasizing that pre-existing metabolic and hepatic conditions exacerbate vulnerability to COVID-19. The presence of multiple comorbidities (especially ≥3) was associated with higher mortality rates (33.3%), reinforcing that the burden of chronic diseases amplifies risk. Interestingly, obesity alone showed no significant association (OR: 1.12), suggesting its impact may be mediated by other concurrent conditions, such as diabetes. In the present analyses, private hospital data revealed a male predominance among COVID-19 patients (59.9%), with a 12.5% mortality rate in this group. Comorbidities were prevalent, including type 2 diabetes (26.3%) and obesity (22.4%), demonstrating a dose-dependent relationship between comorbidity burden and mortality risk (*P*<0.001). This pattern was corroborated by national DataSUS records, which showed similar male predominance (57.3%) with hypertension as the most frequent comorbidity, and critical clinical markers included hypoxemia (SpO_2_ ≤94% in 68.3%), ICU admission (54.0%), and metabolic comorbidities (33.3% diabetes, 6.9% obesity). The observed gender disparity aligns with established biological and epidemiological evidence: (1) occupational exposures increase community transmission risk among men^27^; (2) androgen-regulated ACE2 overexpression enhances viral cellular entry^28^; and (3) X-chromosome hemizygosity in males prevents compensatory ACE2 modulation seen in heterozygous females^29^.

Despite a low overall mortality rate, our findings showed that most patients were admitted to the intensive care unit (ICU), where the risk of death was higher-likely reflecting greater disease severity. The large volume of critically ill patients requiring ICU care during this peak pandemic period may have contributed to these outcomes, as overwhelmed systems faced unprecedented demands. In subsequent waves of COVID-19, the rollout of vaccination significantly improved the prevention of ICU admissions^30^. Several analyses identified a significant association between ICU admission and improved survival, suggesting that timely access to intensive care contributes to better outcomes. At that time, private hospitals had greater ICU availability, which may have played a role in reducing mortality rates^31^.

In the present analysis the in-hospital mortality rate was 10.8% from 50-private hospitals in Brazil in accordance with DataSUS (SARS database) that shows 3.7-fold mortality difference between public (33.0%) and private hospitals (8.96%, *P*<0.001) and these findings reflect Brazil’s fragmented healthcare system, consistent with Marcolino et al.^9^ ^,^ ^31^ Structural factors-including ICU bed shortages, delayed presentations, and pharmacologic disparities (e.g., hydroxychloroquine use)-likely drove this gap^9^ ^,^ ^32^ ^,^ ^33^. Castro et al.^34^ reported a mortality rate of 30.7% up to December 2020, while Ranzani et al.^35^ reported a rate of 38% between February and August, 2020. A retrospective cohort study of COVID-19 hospitalizations between March and October 2020 reported a mortality rate of 21.7%^36^.

The first wave of the COVID-19 pandemic in Brazil faced a combination of political, economic, and social challenges that also contributed to the high mortality rates. Regarding factors that may have contributed to these death rates, since March 2020, there has been a lack of effective national coordination^32^. Misinformation, increased unemployment, and decreased income, combined with difficulties in adhering to social isolation and limited access to health services, exacerbated the impacts of the pandemic^32^.

The significant mortality disparity between public and private hospitals likely reflects structural inequities in Brazil’s healthcare system. Public institutions face systemic challenges including underfunding, overcrowding, and resource limitations that delay care and compromise data quality, while private hospitals offer better-equipped ICUs, favorable staff-to-patient ratios, and earlier diagnostic capabilities^33^. This infrastructure gap results in public hospital patients presenting at later disease stages with higher acuity, whereas private facilities can provide more timely interventions. Notably, the observed differences may be further compounded by under-documentation of comorbidities in overwhelmed public hospitals, potentially masking true risk differential.

Several limitations should be acknowledged, including the retrospective nature of the analysis, potential selection bias due to overrepresentation of private hospital data, and insufficient granularity regarding therapeutic interventions. Additionally, the lack of histopathological liver specimens precludes a definitive assessment of SARS-CoV-2-mediated hepatotoxicity. Specific vaccine impacts and possible adverse events were not evaluated in this study. Finally, the challenges in maintaining accurate and complete records during the pandemic’s high-demand period likely contributed to missing information, potentially affecting the robustness of the conclusions. The under-reporting of cases and deaths due to insufficient diagnostic testing and contact tracing further adds to the uncertainty regarding the actual burden of COVID-19 during the first wave in Brazil.

This study highlights the critical lessons for managing future COVID-19 surges or similar viral outbreaks, particularly in resource-unequal settings: (1) high-risk populations (p. ex. chronic liver disease and malnutrition) require prioritized screening given their elevated mortality risk; (2) AST/ALT monitoring at admission enables early identification of patients likely to deteriorate; (3) gastrointestinal symptoms (diarrhea) should trigger vigilance for milder or severe progression; (4) the 3.7-fold higher mortality in public versus private hospitals demands systemic reforms, including equitable resource distribution and standardized protocols for nutritional support and endoscopic management of bleeding complications; and (5) further studies must validate these findings and explore strategies. These insights emphasize the urgent need to integrate comorbidity monitoring, hepatic biomarkers, and healthcare equity into pandemic preparedness.

## CONCLUSION

This study not only delineates COVID-19’s hepatic and gastrointestinal impact - identifying the AST/ALT ratio, hypoalbuminemia, and diarrhea as critical prognostic markers - but also provides vital lessons for future pandemic preparedness. The mortality disparities between public and private hospitals underscore the life-saving importance of equitable resource allocation during health crises. Moving forward, three key measures should be prioritized: (1) integrating hepatic monitoring into outbreak response protocols, (2) establishing early warning systems that use gastrointestinal manifestations as potential severity markers (3) addressing structural healthcare inequalities that exacerbated outcomes during COVID-19. These evidence-based strategies, drawn from our pandemic experience, could significantly improve outcomes in future infectious disease emergencies, particularly for high-risk populations.

## Data Availability

The research data are presented within the article itself (available in the Results section, Tables 1, 2, 3, and Figure 1).
